# Genome-wide SNP discovery and core marker sets for assessment of genetic variations in cultivated pumpkin (*Cucurbita* spp.)

**DOI:** 10.1038/s41438-020-00342-9

**Published:** 2020-08-01

**Authors:** Nam Ngoc Nguyen, Minkyung Kim, Jin-Kee Jung, Eun-Jo Shim, Sang-Min Chung, Younghoon Park, Gung Pyo Lee, Sung-Chur Sim

**Affiliations:** 1 Department of Bioresources Engineering, Sejong University, Seoul, 05006 South Korea; 2 Seed Testing and Research Center, Korea Seed & Variety Service, Gimcheon, 39660 South Korea; 3 Department of Life Sciences, Dongguk University, Seoul, 04620 South Korea; 4 Department of Horticultural Bioscience, Pusan National University, Miryang, 50463 South Korea; 5 Department of Plant Science and Technology, Chung-Ang University, Ansung, 17546 South Korea; 6 Plant Engineering Research Institute, Sejong University, Seoul, 05006 South Korea

**Keywords:** Plant breeding, Genetic markers

## Abstract

Three pumpkin species *Cucurbita maxima*, *C. moschata*, and *C. pepo* are commonly cultivated worldwide. To identify genome-wide SNPs in these cultivated pumpkin species, we collected 48 F_1_ cultivars consisting of 40 intraspecific hybrids (15 *C. maxima*, 18 *C. moschata*, and 7 *C. pepo*) and 8 interspecific hybrids (*C. maxima* x *C. moschata*). Genotyping by sequencing identified a total of 37,869 confident SNPs in this collection. These SNPs were filtered to generate a subset of 400 SNPs based on polymorphism and genome distribution. Of the 400 SNPs, 288 were used to genotype an additional 188 accessions (94 F_1_ cultivars, 50 breeding lines, and 44 landraces) with a SNP array-based platform. Reliable polymorphisms were observed in 224 SNPs (78.0%) and were used to assess genetic variations between and within the four predefined populations in 223 cultivated pumpkin accessions. Both principal component analysis and UPGMA clustering found four major clusters representing three pumpkin species and interspecific hybrids. This genetic differentiation was supported by pairwise *F*_st_ and Nei’s genetic distance. The interspecific hybrids showed a higher level of genetic diversity relative to the other three populations. Of the 224 SNPs, five subsets of 192, 96, 48, 24, and 12 markers were evaluated for variety identification. The 192, 96, and 48 marker sets identified 204 (91.5%), 190 (85.2%), and 141 (63.2%) of the 223 accessions, respectively, while other subsets showed <25% of variety identification rates. These SNP markers provide a molecular tool with many applications for genetics and breeding in cultivated pumpkin.

## Introduction

Pumpkin (*Cucurbita* spp.; 2*n* = 2*x* = 40) is a major crop in the *Cucurbitaceae* family including cucumber, melon, and watermelon. The *Cucurbita* genus consists of at least 12 diverse species; three major cultivated species are *C. maxima* Duchesne, *C. moschata* Duchesne, and *C. pepo* L.^1^. The cultivated varieties in these species produce thicker, more highly colored, and less fibrous fruit flesh relative to wild species^2^. Pumpkin provides an excellent source of pro-vitamin A, carotenoids, sugars, and minerals^3,4^. In addition, the world production of pumpkin including squash and gourd exceeded 27.6 million tons from 2.04 million ha in 2018^5^. Due to the nutritional and economic value of pumpkin, breeders have made many efforts to develop new varieties in public and private breeding programs. Therefore, plant variety protection (PVP) is important to prevent unauthorized use of new varieties and support breeding activities^6^.

The International Union for the Protection of New Varieties of Plant (UPOV) has harmonized PVP systems among 76 member countries and organizations (as of February 2020). Within this PVP system, a new variety must have distinctness, uniformity, and stability (DUS) to be eligible for registration and protection. UPOV provides a total of 331 guidelines for DUS tests in crop species (as of February 2020). Current DUS testing is mainly based on phenotypic evaluations during two growing seasons, and is labor-intensive, time-consuming, and environment-sensitive^7^. Therefore, the biochemical and molecular techniques (BMT) working group of UPOV have suggested models for the application of molecular markers in variety registration^8,9^. Recent advances in high-throughput genotyping technology have made molecular markers a more attractive option for supplementing or even replacing phenotype-based DUS testing^7,10^.

Molecular markers, especially DNA markers, are an effective tool to explore genetic variations in crop species. Of these markers, simple sequence repeats (SSRs) have been commonly used for DNA fingerprinting and genetic diversity assessment due to advantages such as co-dominant and multi-allelic natures^11–17^. However, SSR markers are not suitable for high-throughput genotyping with a large number of markers. Single nucleotide polymorphisms (SNPs) are amenable to automation for high-throughput and cost-effective genotyping. Next-generation sequencing (NGS) technologies have accelerated the identification of genome-wide SNPs, making SNPs ideal to many applications in plant breeding^18^. In pumpkin, NGS-based transcriptome sequencing of *C. pepo* found over 9,000 SNPs^19^. A total of 8,660 SNPs were also identified from genotyping by sequencing (GBS) in the F_2_ population (*n* = 186), which were derived from two inbred lines of *C. maxima*^20^. These SNPs were used to construct high-density genetic maps and to detect QTL associated with a dwarf vine. In addition, the GBS-based SNP studies were conducted in the pumpkin species (*C. pepo*, *C. moschata*, and *C*. *okeechobeensis* subsp. *martinezii*) and the resulting SNPs were used to map loci associated with powdery mildew resistance and fruit-related traits^21,22^. Recently, the *Cucurbita* genomes were assembled in *C. maxima*, *C. moschata*, and *C. pepo*^23,24^. For *C. pepo*, the genomes of seven morphotypes were also studied by resequencing with an average of 33.5x coverage^25^. These genome resources have accelerated genome-wide SNP discovery in cultivated pumpkin.

Although a large number of SNPs were previously identified in pumpkins, this genomic resource is limited to investigation of genetic variations and variety identification in cultivated pumpkin germplasm including commercial F_1_ cultivars. Therefore, we generated genome-wide SNPs with a GBS approach in a collection of 48 commercial F_1_ cultivars representing intraspecific hybrids for each of three pumpkin species (*C. maxima*, *C. moschata*, and *C. pepo*) and interspecific hybrids (*C. maxima* × *C. moschata*). Of these, 288 SNPs were used to genotype an additional collection of 188 accessions with the Fluidigm platform. The genetic variations between and within pumpkin populations were assessed based on these SNP markers. In addition, several subsets of SNP markers were generated for variety identification in commercial F_1_ cultivars. These SNP markers are a useful resource for developing a cost-effective and rapid DNA-based system for DUS testing and thus benefit breeders by protecting their ownerships of new pumpkin varieties.

## Results

### Genome-wide SNP discovery in commercial F_1_ pumpkin cultivars

**Fig. 1:**
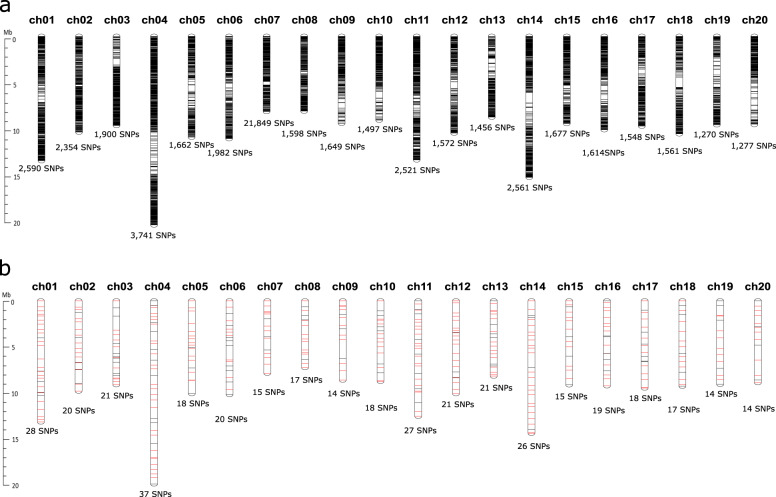
SNP distribution on 20 pumpkin chromosomes. Horizontal lines on each chromosome indicate physical map positions of 37,869 confident SNPs from genotyping by sequencing (GBS) in the 48 commercial F_1_ cultivars (**a**) and a subset of 400 SNPs (**b**). The red lines indicate the 288 SNPs used for the Fluidigm assay. The physical positions of SNPs are based on the *C. maxima* genome assembly^24^

The sequencing of GBS libraries for the 48 F_1_ cultivars generated a total of 389.9 million reads ranging from 2.4 million to 13.2 million per cultivar with an average of 8.1 million (Table 1). All of these reads represented 39.4 Gb that is 102x coverage for the genome assembly (386.8 Mb) of *C. maxima*^24^. The 381.6 million reads (97.9%) showed expected barcodes and 953,780 tags were mapped to the *C. maxima* genome. The TASSEL-GBS pipeline detected a total of 232,256 variants including 202,722 SNPs (Table 1). Of these, we obtained 37,869 bi-allelic SNPs with >5% of minor allele frequency and <10% of missing data. These SNPs were unevenly distributed on 20 chromosomes ranging from 1,270 to 3,741 SNPs per chromosome (Fig. 1a). Furthermore, the number of SNP in four predefined populations varied from 26,707 (*C. maxima* × *C. moschata*) to 34,869 (*C. pepo*) (Table 2). We detected 23,703 transition SNPs (62.6%) and 14,166 transversion SNPs (37.4%) in the collection of 48 F_1_ cultivars. Similarly, the number of transition SNPs was ~1.7 times higher relative to transversion SNPs in all four populations (Table 2). Two transition types (A/G and C/T) showed similar numbers, while the number of the A/T transversion type was higher than the other types (A/C, G/T, and C/G).

**Table 1:** Summary of genotyping by sequencing (GBS) in the 48 F_1_ pumpkin cultivars

*Illumina pair-end sequencing*	
No. of raw reads	389,907,450
Average length of raw reads (bp)	100
Total length of raw reads (Gb)	39.4
*TASSEL-GBS analysis*	
No. of good barcoded reads	381,650,314
No. of tags	25,206,620
No. of tags with minimum counts of five	1,357,210
No. of mapped tags	953,780
No. of variants	232,256
No. of total SNPs	202,722
No. of filtered SNPs^a^	37,869

^a^Bi-allelic SNPs across the 20 pumpkin chromosomes passed three criteria: minor allele frequency of >5%, missing data rate of <10%, and minimum depth of 5x

**Table 2:** GBS-based SNP calls in the four predefined populations of the 48 F_1_ pumpkin cultivars

Predefined population	*C. maxima*	*C. moschata*	*C. pepo*	*C. maxima* × *C. moschata*	All combined
Sample size	15	18	7	8	48
No. of SNPs	27,317	28,342	34,869	26,707	37,869
Transition SNPs	17,232 (63.1%)	17,816 (62.9%)	21,831 (62.6%)	16,790 (62.9%)	23,703 (62.6%)
A/G	8,549	8,835	10,853	8,310	11,817
C/T	8,683	8,981	10,978	8,480	11,886
Transversion SNPs	10,085 (36.9%)	10,526 (37.1%)	13,038 (37.4%)	9,917 (37.1%)	14,166 (37.4%)
A/T	3,085	3,174	3,932	2,995	4,257
A/C	2,358	2,486	3,020	2,344	3,302
G/T	2,418	2,526	3,089	2,372	3,378
C/G	2,224	2,340	2,997	2,206	3,229

The polymorphic information content (PIC) values of 37,869 confident SNPs were calculated based on polymorphisms in the 48 F_1_ cultivars. The 24,684 SNPs with ≥0.3 PIC values were used to select core sets of SNP markers for variety identification. First, a subset of 400 SNPs was filtered based on their physical positions relative to the *C. maxima* genome. These SNPs were distributed across 20 chromosomes with the average intervals ranging from 0.41 Mb (chromosome 13) to 0.69 Mb (chromosome 19) (Fig. 1b). Based on the *C. maxima* annotation^24^, 287 SNPs (71.7%) were derived from coding sequences, consisting of 126 non-synonymous and 161 synonymous SNPs (Table 3). The remaining 113 SNPs (28.2%) were non-coding sequence variants. Of these, 47 and 24 SNPs were upstream and downstream gene variants, respectively, while the other 42 SNPs were from UTRs, introns, splice sites, and intergenic regions (Table 3).

**Table 3:** The subsets of genome-wide SNPs for validation and core marker selection

Class^a^		No. of SNP^b^
Coding sequence variant	Non-synonymous variant	126 (109)
	Synonymous variant	161 (105)
Non-coding sequence variant	Upstream gene variant	47 (29)
	Downstream gene variant	24 (16)
	UTR variant	11 (8)
	Intron variant	14 (11)
	Splice region variant	9 (5)
	Intergenic variant	8 (5)
Total		400 (288)

^a^This is based on the annotation of the *C. maxima* genome^24^

^b^Number in the parentheses indicates SNPs used for the Fluidigm assay in an additional collection of 188 pumpkin accessions

### SNP chip-based genotyping for validation

We used 288 of 400 SNPs to genotype an additional collection of 188 pumpkin accessions in the Fluidigm assay. The 174 accessions (92.6%) were genotyped with call rates >90%. One accession of *C. pepo* (breeding line ‘Zhdana’) showed a call rate of 83.8%. Since the other 13 accessions (4 F_1_ cultivars and 9 landraces) showed call rates of 36.6–62.0%, these were excluded from further analyses. In the 174 accessions, 224 of 288 SNPs (77.8%) were polymorphic and 13 SNPs (4.5%) were monomorphic (Table 4). Of these polymorphic SNPs, 165 SNPs (73.7%) were derived from coding sequences and 109 SNPs (48.7%) were non-synonymous. In addition, the genotypes of 19 SNPs were undetermined due to ambiguous clustering patterns and 32 SNPs showed no call.

**Table 4:** Polymorphism of 288 SNP markers in the collection of additional 188 pumpkin accessions used for the Fluidigm assay

Class	No. of markers	Percentage (%)
Polymorphic	224	77.8
Monomorphic	13	4.5
Undetermined^a^	19	6.6
No call	32	11.1
Total	288	100.0

^a^Polymorphism detected but ambiguous genotype calls or high percentage of missing data (≥30%)

**Fig. 2:**
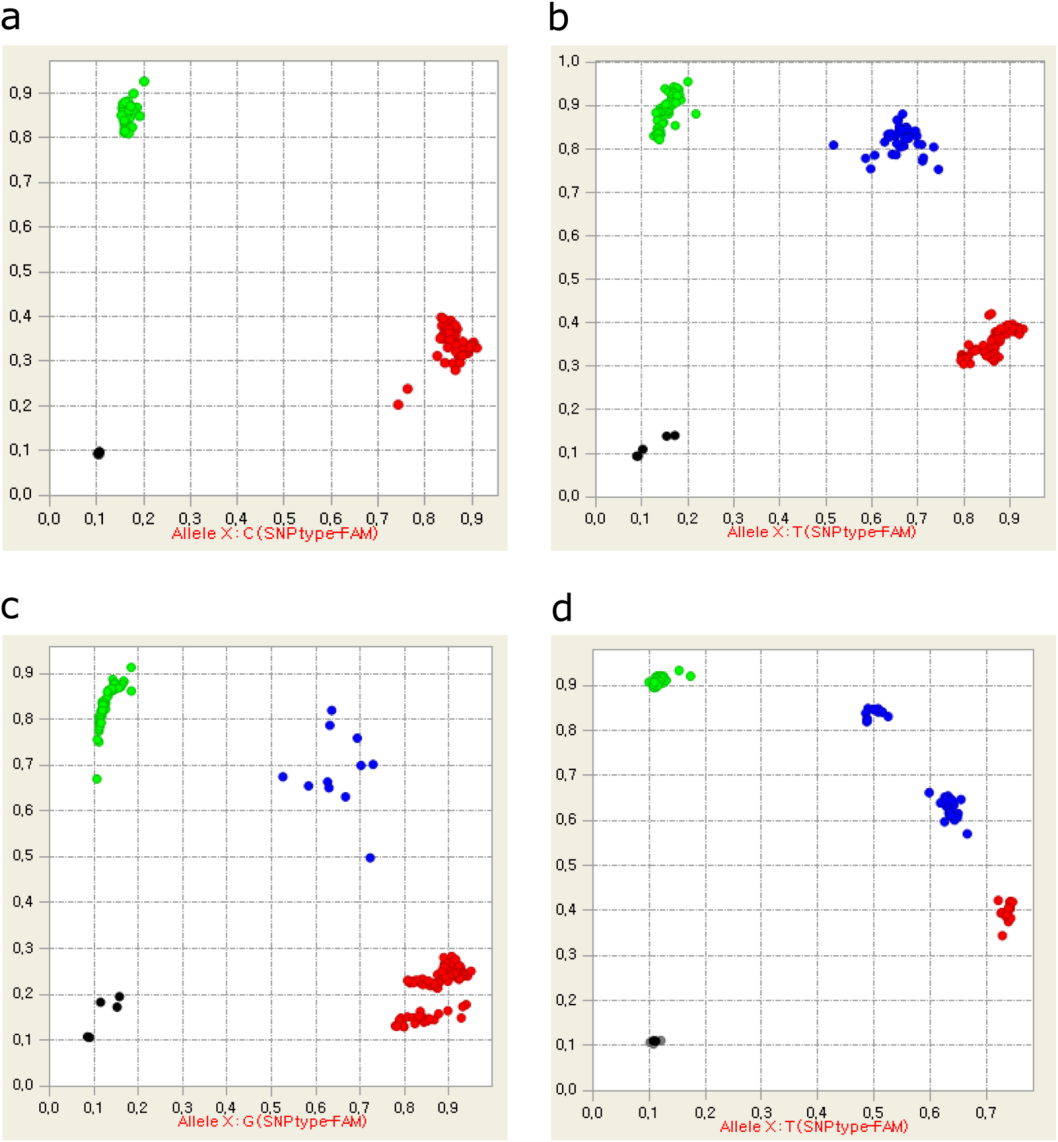
Scatter plots for SNP calling in the Fluidigm assay. Each color code in the plots presents one of three genotypes: homozygote of allele 1 (red), homozygote of allele 2 (green), and heterozygote (blue). Normal clustering patterns are shown with clear separation between three genotypes (**a**, **b**). The secondary SNPs on the primer annealing sites cause unusual clustering patterns (**c**, **d**)

Most of the SNPs showed two or three clusters corresponding to two homozygous genotypes (XX and YY) and a heterozygous genotype (XY) (Fig. 2a, b). However, we observed different clustering patterns for several SNPs (Fig. 2c, d). For example, the SNP marker ‘S14_2246878’ showed two clusters for a homozygous genotype (Fig. 2c). Similarly, the same heterozygous genotypes were separated into two clusters for the SNP marker ‘S14_9835352’ (Fig. 2d). The Sanger sequencing for the flanking sequences of these SNPs detected additional SNPs in the binding sites of specific target amplification and locus specific primers (Fig. S1a and S1b). These secondary SNPs could lead to inaccurate amplification for the target alleles and thus result in these clustering patterns.

### Genetic variations between and within cultivated pumpkin populations

**Fig. 3:**
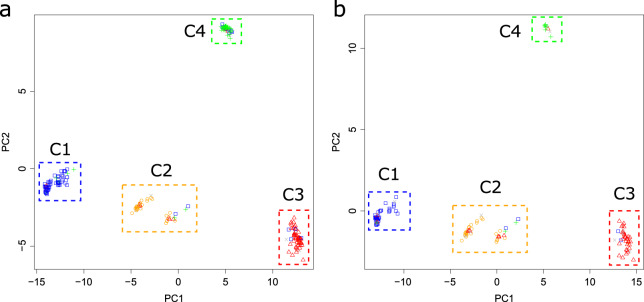
Principal component analysis (PCA) of the cultivated pumpkin accessions. Two principal components (PC 1 and PC 2) based on 224 SNP markers are shown in the plots for all 223 cultivated pumpkin accessions (**a**) and a subset of 138 F_1_ cultivars (**b**). The color codes indicate *C. maxima* (blue), *C. moschata* (red), *C. maxima* x *C. moschata* hybrid (orange), *C. pepo* (green), and unknown (gray) accessions

The genotypic data of 224 SNP markers were used to investigate genetic variations in the 223 pumpkin accessions used for GBS and Fluidigm assays. This collection consisted of 73 *C. maxima*, 63 *C. moschata*, 45 *C. pepo*, 31 interspecific hybrids (*C. maxima* × *C. moschata*), and 11 unknown accessions. In PCA, the 223 pumpkin accessions were divided into four major clusters using the first two principal components (PC1 and PC2), which explained 65.9% and 15.3% of the total variance, respectively (Fig. 3a). Of the 73 *C. maxima* accessions, 59 (38 F_1_ cultivars, 12 breeding lines, and 9 landraces) were grouped with two *C. moschata* (all F_1_ cultivars), five *C. pepo* (one F_1_ cultivar and four breeding lines), and two unknown accessions (all F_1_ cultivars) in cluster 1. We found 30 of 31 interspecific hybrids in cluster 2, which also included 10 intraspecific hybrids (two *C. maxima*, six *C. moschata*, and two *C. pepo*) and three unknown F_1_ cultivars. These accessions in cluster 2 were further divided into two sub-clusters based on PC1 (Fig. 3a). One sub-cluster contained seven interspecific and seven intraspecific hybrids, while another sub-cluster contained 23 interspecific and six intraspecific hybrids (Table S1). In cluster 3, 53 of the 63 *C. moschata* accessions were found with four *C. maxima* (two F_1_ cultivars and two breeding lines), one interspecific hybrid, and four unknown (all F_1_ cultivars) accessions. Cluster 4 consisted of 38 *C. pepo*, eight *C. maxima* (five breeding lines and three landraces), and two *C. moschata* (one F_1_ cultivar and one breeding line), and two unknown (all F_1_ cultivars) accessions. Most of the pumpkin accessions were separated according to their predefined populations based on *a priori knowledge* using PC1 and PC2. However, we found no obvious genetic differentiation among F_1_ cultivars, breeding lines, and landraces (Fig. 3a, b). The F_1_ cultivars were found in all four clusters, while breeding lines and landraces were distributed across clusters 1, 3, and 4.

**Fig. 4:**
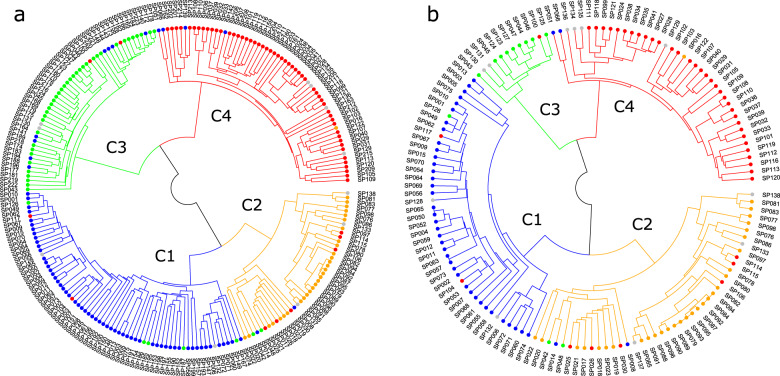
UPGMA dendrograms of the cultivated pumpkin accessions. The Euclidian genetic distances for the dendrograms are calculated using the 224 SNP markers in all 223 cultivated pumpkin accessions (**a**) and a subset of 138 F_1_ cultivars (**b**). The color codes indicate *C. maxima* (blue), *C. moschata* (red), *C. maxima* x *C. moschata* hybrid (orange), *C. pepo* (green), and unknown (gray) accessions

The UPGMA dendrogram based on Euclidian genetic distances showed four clusters in the 223 pumpkin accessions (Fig. 4a). Moreover, the 138 F_1_ cultivars were also separated into four clusters as shown in PCA (Fig. 4b). Only four of the 223 accessions (three F_1_ cultivars and one landrace) were differently clustered between the UPGMA and PCA methods, indicating a consistent result (Table S1). The magnitude and significance of genetic differentiation between the four predefined populations were measured using pairwise *F*_st_ and Nei’s genetic distance (*D*). For this analysis, we excluded the 11 unknown accessions. The four populations were all significantly differentiated by pairwise *F*_st_ at *P* < 0.001 (Table 5). We found the highest level of genetic differentiation between *C. maxima* and *C. moschata* populations (*F*_st_ = 0.63 and *D* = 0.58). The *C. pepo* population was separated from *C. maxima* (*F*_st_ = 0.49 and *D* = 0.30) and *C. moschata* (*F*_st_ = 0.46 and *D* = 0.24) populations. The pairwise estimates of *F*_st_ and *D* suggested that the interspecific hybrids were more similar to *C. maxima* (*F*_st_ = 0.21 and *D* = 0.12) than *C. moschata* (*F*_st_ = 0.41 and *D* = 0.30) (Table 5).

**Table 5:** Nei’s genetic distance and pairwise estimates of *F*_st_ between the pumpkin populations based on 224 SNP markers

Predefined population^a^	*C. maxima*	*C. maxima* × *C. moschata*	*C. moschata*	*C. pepo*
*C. maxima*		0.12^b^	0.58	0.30
*C. maxima* × *C. moschata*	0.21*		0.30	0.22
*C. moschata*	0.63*	0.41*		0.24
*C. pepo*	0.49*	0.37*	0.46*	

^a^The 11 unknown accessions were excluded

^b^Nei’s standard genetic distance corrected for sample size^45^ (upper right diagonal) and pairwise estimates of *F*_st_^41^ (lower left diagonal) between populations. *P*-values were calculated by 10,000 permutations with a Bonferroni correction

*P < 0.001

Allelic richness (A), expected heterozygosity (He), and PIC were used to investigate levels of genetic diversity within each of the predefined populations (Table 6). The *C. maxima* × *C. moschata* hybrids showed the highest estimates of these descriptive statistics (A = 1.98, He = 0.42, and PIC = 0.32), while the *C. pepo* population showed the lowest estimates (A = 1.77, He = 0.18, and PIC = 0.15). Similar levels of genetic diversity were found in the *C. maxima* (A = 1.90, He = 0.27, and PIC = 0.22) and *C.moschata* (A = 1.90, He = 0.22, and PIC = 0.19) populations (Table 6).

**Table 6:** Descriptive statistics for genetic diversity within the pumpkin populations based on 224 SNP markers

Predefined population^a^	Sample size	A^b^	He^c^	PIC^d^
*C. maxima*	73	1.90	0.27	0.22
*C. moschata*	63	1.90	0.22	0.19
*C. pepo*	45	1.77	0.18	0.15
*C. maxima* × *C. moschata*	31	1.98	0.42	0.32
Total	223	1.99	0.44	0.35

^a^The 11 unknown accessions were excluded

^b^Allelic richness^43,44^

^c^Expected heterozygosity corrected for sample size^45^

^d^Polymorphism information content^37^

### Core SNP markers for variety identification

The 224 SNP markers differentiated 211 (94.6%) of the 223 pumpkin accessions including all inbred accessions and 126 of 138 F_1_ cultivars (Fig. 4a, b). From these markers, we selected 192 SNP markers as a core set for variety identification based on their polymorphisms. These core markers were effective in identifying 204 (91.5%) of the 223 accessions. The remaining 19 accessions, which were not separated by the 192 SNP markers, consisted of 11 interspecific hybrids and 8 intraspecific hybrids (4 *C. maxima* and 4 *C. moschata*). Interestingly, all 45 *C. pepo* accessions including F_1_ were distinct using this core set of SNP markers (Fig. 4a, b). Four additional subsets of 96, 48, 24, and 12 SNPs were generated from the 192 SNP markers to evaluate their performance for variety identification (Figs. S2 and S3). The 92 and 48 SNP markers detected genetic variations to distinguish 190 (85.2%) and 141 (63.2%) of the 223 pumpkin accessions, while the 24 and 12 SNP markers identified 54 (24.2%) and 11 (4.9%) accessions, respectively (Fig. S2). The 92 and 48 marker sets revealed four clusters representing the predefined populations using PC1 and PC2 as the 192 marker set (Fig. S3). Although accessions in each cluster were loosely grouped, the 24 marker set was also able to detect these four clusters. Therefore, the 24 marker set can be used for the pre-identification of pumpkin varieties based on species.

## Discussion

Next-generation sequencing (NGS) technologies have led to rapid SNP discovery and high-throughput genotyping. As an NGS-based method, genotyping by sequencing (GBS) is a cost-effective approach based on genome complexity reduction for identifying genome-wide SNPs^26^. Therefore, GBS have been commonly used as a powerful tool for high-resolution genetic mapping, genome-wide association study, and genetic diversity analysis in crop species^20,27–29^. In this study, a total of 37,869 confident SNPs were generated using GBS in the collection of 48 F_1_ pumpkin cultivars representing three main *Cucurbit* species (*C. maxima*, *C. moschata*, and *C. pepo*) and interspecific hybrids (*C. maxima* × *C. moschata*). In addition, 26,707–34,869 SNPs were found in each of these four populations. Previous studies in pumpkins reported relatively small numbers of SNPs using inbred accessions. Blanca et al.^19^ identified 9,043 filtered SNPs between two *C. pepo* subspecies using NGS-based transcriptome sequencing. The GBS study generated 8,660 SNPs in the F_2_ population of *C. maxima*^20^. We also found similar percentages of transition (~63%) and transversion (~37%) SNPs relative to those in the study of Blanca et al.^19^. The genome-wide SNPs from the present study may be biased to *C. maxima* because the GBS reads of 48 F_1_ cultivars were mapped to the *C. maxima* genome assembly for SNP discovery. Therefore, the GBS reads are deposited in the Sequence Read Archive (SRA) of NCBI (PRJNA633011) for a customized SNP identification with the *C. moschata* or *C. pepo* genomes. Our results contribute to developing a large SNP collection that is a useful resource to investigate genetic variations in three major pumpkin species.

A subset of 288 SNPs with ≥0.3 PIC values was used to genotype the 188 pumpkin accessions (94 F1 cultivars, 50 breeding lines, and 44 landraces) in the Fluidigm assay. Of these, 224 SNPs (77.8%) showed clear polymorphism in this collection. With these SNP markers, the 223 pumpkin accessions including 48 F_1_ cultivars used for GBS were separated into four clusters in both PCA and UPGMA dendrogram. Most of the accessions in each cluster were derived from *C. maxima*, *C. moschata*, *C. pepo*, or *C. maxima* × *C. moschata* populations. The pairwise *F*_st_ and Nei’s genetic distance also indicated significant genetic differentiation between these four populations. Similar genetic relationships between these *Cucurbit* species were also previously found using SSR markers^16,30,31^. In addition, the sub-division in the *C. maxima* × *C. moschata* accessions suggests that the interspecific hybrids used in this study are differentiated from two different genetic backgrounds. These results demonstrate that the SNP markers are a powerful tool to detect species-specific loci and/or alleles in discriminating *Cucurbit* species.

Both *C. maxima* (*n* = 73) and *C. moschata* (*n* = 63) populations displayed higher estimates of allelic richness, expected heterozygosity, and PIC than the *C. pepo* population (*n* = 45). In the study of Gong et al.^30^, the *C. pepo* accessions showed the highest levels of genetic diversity among these three species, despite having a smaller number of accessions (18 *C. maxima*, 20 *C. moschata*, and 7 *C. pepo*). Cultivated *C. pepo*, which is known to have a great diversity of morphology, consisted of two subspecies: ssp.*pepo* (Pumpkin, Vegetable Marrow, Cocozelle, and Zucchini) and ssp.*ovifers* (Acorn, Scallop, Crookneck, and Straightneck)^32,33^. In the PCA analysis, our *C. pepo* accessions were tightly aggregated in a cluster relative to the other two species, suggesting that the *C. pepo* population represents few morphotypes including Zucchini. Therefore, it is possible that this discrepancy in genetic diversity of *C. pepo* is due to the sampling of accessions.

To recognize breeder’s intellectual property rights, new varieties must satisfy three criteria in the plant variety protection (PVP) system: distinctness, uniformity and stability (DUS). The current DUS testing based on phenotypic evaluation involves laborious and time-consuming tasks. Therefore, a DNA-based system with molecular markers has been considered as an alternative to improve the efficiency and accuracy of DUS testing^7,10^. With NGS technologies and high-throughput genotyping platforms, genome-wide SNP markers have been widely used across numerous applications in crop species^18^. The core set of 192 SNP markers in this study were sufficient in detecting genetic variations for identification of all 85 pumpkin inbred accessions. For the collection of 138 commercial F_1_ cultivars, 119 accessions were identified using these SNP markers. The unidentified 19 F_1_ cultivars, which consist of 11 interspecific hybrids (*C. maxima* × *C. moschata*) and 8 intraspecific hybrids (4 *C. maxima* and 4 *C. moschata*), are likely to have narrow genetic bases. Kong et al.^31^ also found similar genetic backgrounds between the commercial F_1_ cultivars of *C. maxima* x *C. moschata*. A possible explanation for these reduced genetic bases is to use a few elite inbred lines as parents to develop different F_1_ cultivars in breeding programs. Therefore, variety identification in the F_1_ cultivars with high levels of genetic similarity is often challenging. Our study revealed that the 224 SNP markers identified six more interspecific hybrids relative to the 192 SNP markers, suggesting that increasing the number of SNP markers allows to us distinguish these F_1_ cultivars. Since we identified a total of 26,707 SNPs in the interspecific hybrid population using GBS, it is possible to find additional markers that are effective in detecting minimal genetic variations in unidentified F_1_ cultivars.

Although the core set of 192 SNP markers is a powerful tool for variety identification, their subsets can also be useful in providing additional options for genotyping with different platforms. Of the four subsets, the 96 and 48 SNP markers were able to identify 85.2% and 63.2% of the 223 pumpkin accessions, respectively. The other two subsets of 24 and 12 SNP markers showed low identification rates (24.2% and 4.9%, respectively). This result indicates that the subsets of 96 and 48 SNPs are suitable for pre-screening tests using cost-effective genotyping platforms. The 24 marker set can also be sufficient when detecting species-specific genetic variations between intraspecific hybrids. Thus, these subsets of SNP markers are valuable resources for developing a DNA-based system for PVP in pumpkin.

In conclusion, a large collection of SNPs was generated for three major pumpkin species (*C. maxima*, *C. moschata*, and *C. pepo*) using GBS and commercial F_1_ cultivars. These SNPs contribute to an expansion of genomic resources for both basic and applied researches in cultivated pumpkin. Our results also demonstrate that the core sets of SNP markers are useful for exploring genetic variations between and within the four pumpkin populations representing three species and interspecific hybrids (*C. maxima* × *C. moschata*). Furthermore, these SNP markers provide a rapid and accurate option for variety identification and facilitate development of a DNA-based system for DUS testing in the PVP system. Other applications of these markers include seed purity tests and background selection in breeding programs.

## Materials and methods

### Plant materials and DNA isolation

A total of 48 commercial F_1_ cultivars was collected to identify genome-wide SNPs via genotyping by sequencing (GBS). This collection included 40 intraspecific hybrids of three species (15 *C. maxima*, 18 *C. moschata*, and 7 *C. pepo*) and eight interspecific hybrids (*C. maxima* × *C. moschata*) derived from 25 seed companies (Table S1). We also used an additional collection of 188 germplasm consisting of 94 F_1_ cultivars and 94 inbred accessions (50 breeding lines and 44 landraces) for SNP validation (Table S1). These inbred accessions were collected from the National Agrobiodiversity Center in Rural Development Administration in the Republic of Korea (ROK). Their countries of origin are ROK (13 breeding lines and 8 landraces), the United States (5 breeding lines and 10 landraces), Russia (9 breeding lines and 5 landraces), China (12 breeding lines), Bulgaria (one breeding line and 8 landraces), Turkey (2 breeding lines and 7 landraces), Ukraine (8 breeding lines), and Nepal (6 landraces).

Genomic DNA was extracted from fresh, young leaves using a modified cetyl trimethyl ammonium bromide (CTAB) method^34^. The quality and quantity of DNA was measured using the NanoDrop 1000 spectrophotometer (ThermoFisher Scientific, Wilmington, DE 19810, USA). The final concentration of DNA was adjusted to 50 ng/μL for GBS and Fluidigm assay.

### Genotyping by sequencing (GBS)

GBS libraries of 48 F_1_ cultivars were prepared according to the protocol described by Elshire et al.^26^. The 200 ng of genomic DNA for each cultivar were digested using a methylation-sensitive restriction enzyme, *Ape*KI (NEB, Ipswich, MA, USA). After digestion, the DNA fragments were ligated to different barcode adapters that were assigned to each cultivar. These DNA samples were pooled and amplified by PCR to generate GBS libraries. The libraries were sequenced with the pair-end method in the HiSeq 2500 platform (Illumina Inc., San Diego, CA, USA). For SNP calling, the filtered, high-quality sequencing reads were mapped to the *C. maxima* (Rimu) genome^24^ using the Burrows-Wheeler Alignment (BWA) method^35^ in the TASSEL-GBS pipeline^36^. The resulting bi-allelic SNPs with 5x of minimum depth were filtered based on >5% of minor allele frequency and <10% of missing data for further analysis.

### Fluidigm genotyping with SNP markers

A subset of SNPs was selected based on polymorphism information content (PIC) value and physical position on 20 chromosomes for SNP genotyping with the Fluidigm Juno^TM^ system (Fluidigm, San Francisco, CA, USA) in the 188 pumpkin accessions. The PIC value for each SNP was calculated using the following equation:

$${\mathrm{PIC = 1}} - \mathop {\sum }\limits_{i = 1}^n p_i^2 - \mathop {\sum }\limits_{i = 1}^{n - 1} \mathop {\sum }\limits_{j = i + 1}^n 2p_i^2p_j^2$$

where *n* is the number of alleles and *p*_*i*_ is the frequency of the *i*th allele^37^.

For the Fluidigm SNP genotyping, three types of primers were designed using the 300 bp flanking sequence of each SNP and the D3 Assay Design software (Fluidigm, San Francisco, CA, USA). Both specific target amplification and locus specific primers were used for pre-amplification and two allele specific primers were used for PCR amplification in the Juno 96.96 Genotyping IFC (Integrated Fluidic Circuit). The resulting end-point fluorescence images were analyzed for SNP calling using the Fluidigm SNP genotyping analysis software v4.5.1.

### Data analysis

The genotypic data from both GBS and Fluidigm SNP genotyping were used to investigate genetic variations in the cultivated pumpkin germplasm (Table S2). Principal component analysis (PCA) was performed using the pcaMethods package^38^ as implemented in R^39^. The Euclidean genetic distances were also calculated between pumpkin accessions with the dist function and hierarchical cluster analysis was then conducted using the unweighted pair group method with arithmetic mean (UPGMA) and the hclust function in R. The UPGMA dendrogram was visualized using the R package dendextent^40^. Pairwise *F*_st_^41^ and Nei’s genetic distance were estimated between four predefined populations of the pumpkin collection using the Microsatellite Analyzer (MSA) software v4.05^42^. The *P*-value for the pairwise *F*_st_ was obtained from 10,000 permutations of genotypes and an applied Bonferroni correction. In addition, allelic richness (A)^43,44^ and expected heterozygosity (He)^45^ were calculated for the pumpkin populations using MSA.

## Supplementary information

Figure S1

Figure S2

Figure S3

Table S1

Table S2

## Data Availability

All of the GBS reads of 48 F_1_ pumpkin cultivars generated in this study are deposited in the Sequence Read Archive (SRA) of National Center of Biotechnology Information (NCBI) with the BioProject accession number PRJNA633011 (Release data: 08-30-2020). The genotypic data of SNP markers for the 223 pumpkin accessions are included as supplementary information.
