# Supplementary material for: Genome-wide SNP discovery and core marker sets for assessment of genetic variations in cultivated pumpkin (Cucurbita spp.)
Source: Hortic Res. 2020 Aug 1;7:121. doi: 10.1038/s41438-020-00342-9 (PMC7395168; doi:10.1038/s41438-020-00342-9)
Supplement: Supplementary file 1 — Figure S1 [file 41438_2020_342_MOESM1_ESM.pptx]

## Slide 1
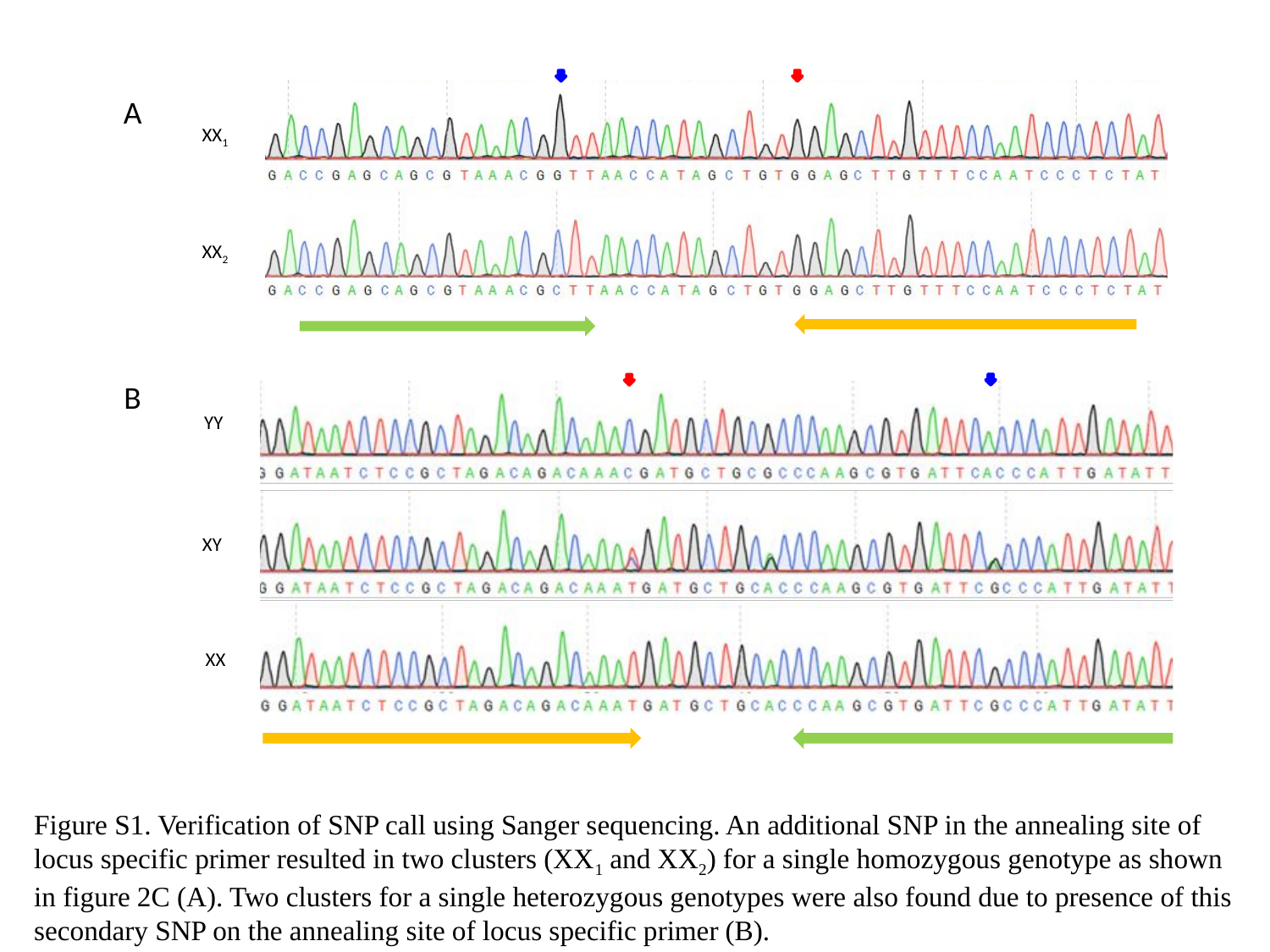

XX1
XX2
A
B
YY
XY
XX
Figure S1. Verification of SNP call using Sanger sequencing. An additional SNP in the annealing site of locus specific primer resulted in two clusters (XX1 and XX2) for a single homozygous genotype as shown in figure 2C (A). Two clusters for a single heterozygous genotypes were also found due to presence of this secondary SNP on the annealing site of locus specific primer (B).
