# Supplementary material for: Genome-wide SNP discovery and core marker sets for assessment of genetic variations in cultivated pumpkin (Cucurbita spp.)
Source: Hortic Res. 2020 Aug 1;7:121. doi: 10.1038/s41438-020-00342-9 (PMC7395168; doi:10.1038/s41438-020-00342-9)
Supplement: Supplementary file 2 — Figure S2 [file 41438_2020_342_MOESM2_ESM.pptx]

## Slide 1
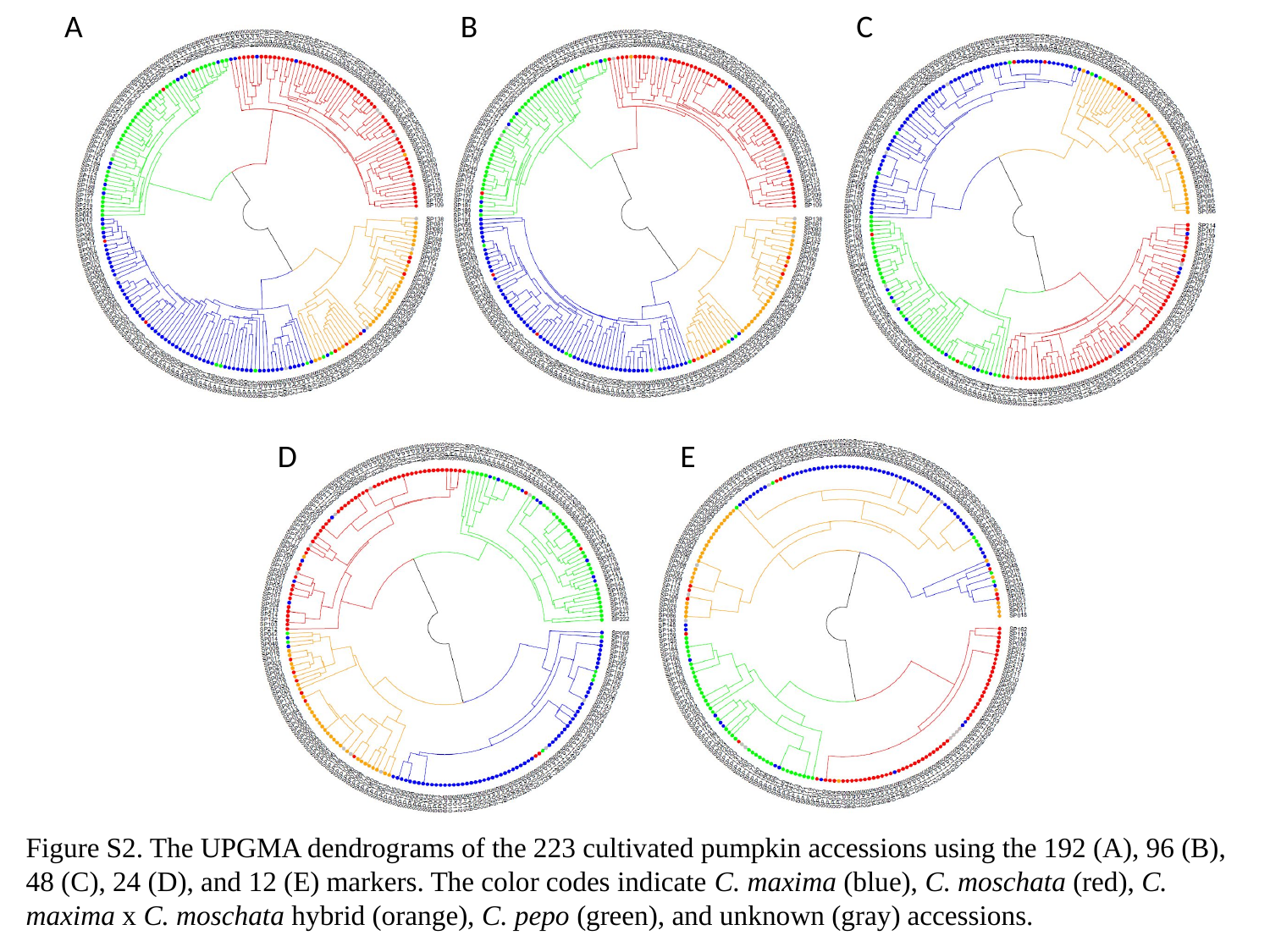

A
B
C
E
D
Figure S2. The UPGMA dendrograms of the 223 cultivated pumpkin accessions using the 192 (A), 96 (B), 48 (C), 24 (D), and 12 (E) markers. The color codes indicate C. maxima (blue), C. moschata (red), C. maxima x C. moschata hybrid (orange), C. pepo (green), and unknown (gray) accessions.
