# Supplementary material for: Genome-wide SNP discovery and core marker sets for assessment of genetic variations in cultivated pumpkin (Cucurbita spp.)
Source: Hortic Res. 2020 Aug 1;7:121. doi: 10.1038/s41438-020-00342-9 (PMC7395168; doi:10.1038/s41438-020-00342-9)
Supplement: Supplementary file 3 — Figure S3 [file 41438_2020_342_MOESM3_ESM.pptx]

## Slide 1
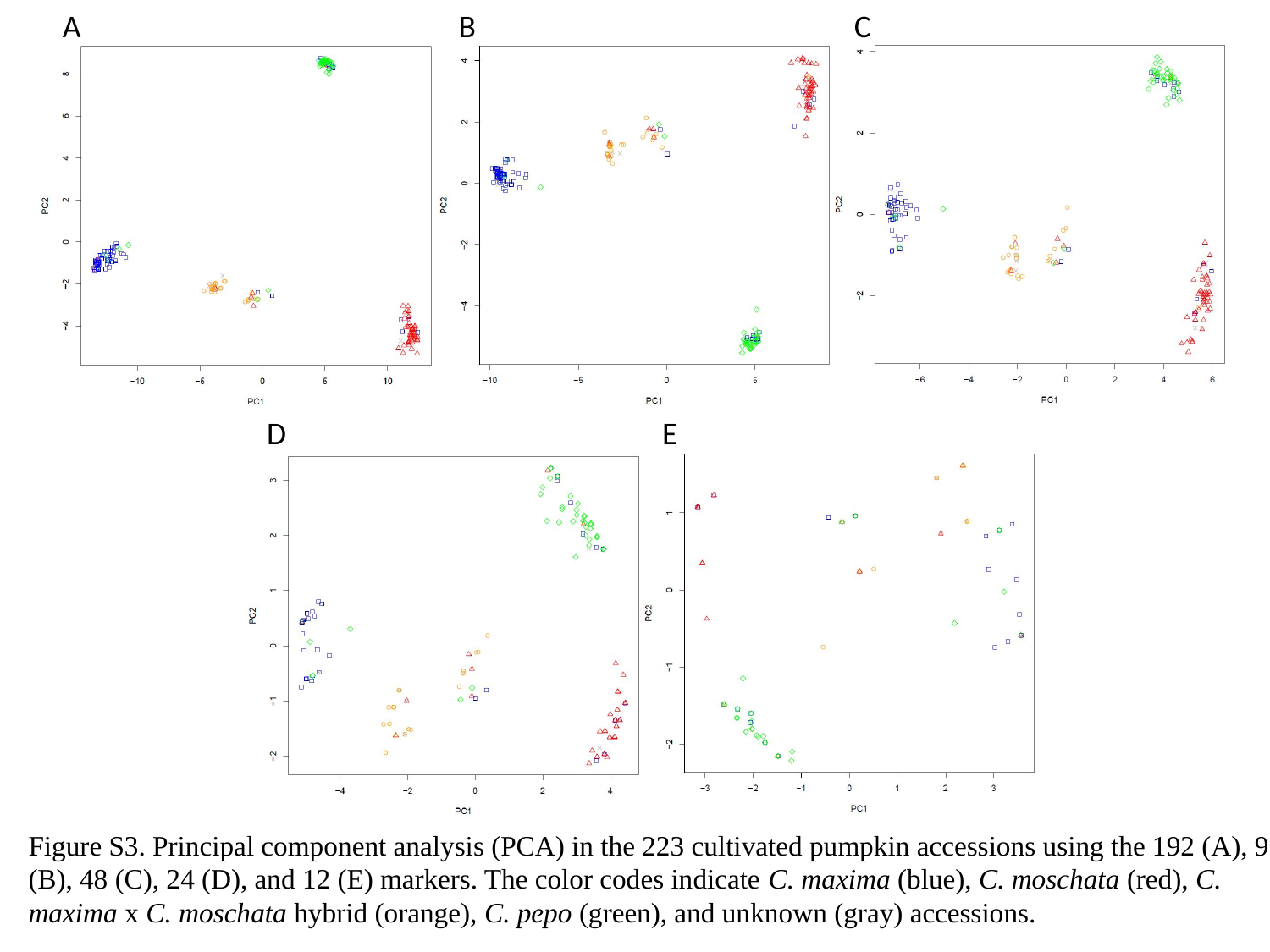

A
B
C
D
E
Figure S3. Principal component analysis (PCA) in the 223 cultivated pumpkin accessions using the 192 (A), 96 (B), 48 (C), 24 (D), and 12 (E) markers. The color codes indicate C. maxima (blue), C. moschata (red), C. maxima x C. moschata hybrid (orange), C. pepo (green), and unknown (gray) accessions.
